# A Reassessment of Genes Modulating Aging in Mice Using Demographic Measurements of the Rate of Aging

**DOI:** 10.1534/genetics.118.300821

**Published:** 2018-02-14

**Authors:** João Pedro de Magalhães, Louise Thompson, Izabella de Lima, Dale Gaskill, Xiaoyu Li, Daniel Thornton, Chenhao Yang, Daniel Palmer

**Affiliations:** Integrative Genomics of Ageing Group, Institute of Ageing and Chronic Disease, University of Liverpool, L7 8TX, United Kingdom

**Keywords:** longevity, meta-analysis, hazard rate, progeria, survival, *Mus musculus*

## Abstract

Many studies have reported genetic interventions that have an effect on mouse life span; however, it is crucial to discriminate between manipulations of aging and aging-independent causes of life extension. Here, we used the Gompertz equation to determine whether previously reported aging-related mouse genes statistically affect the demographic rate of aging. Of 30 genetic manipulations previously reported to extend life span, for only two we found evidence of retarding demographic aging: *Cisd2* and *hMTH1*. Of 24 genetic manipulations reported to shorten life span and induce premature aging features, we found evidence of five accelerating demographic aging: *Casp2*, *Fn1*, *IKK*-β, *JunD*, and *Stub1*. Overall, our reassessment found that only 15% of the genetic manipulations analyzed significantly affected the demographic rate of aging as predicted, suggesting that a relatively small proportion of interventions affecting longevity do so by regulating the rate of aging. By contrast, genetic manipulations affecting longevity tend to impact on aging-independent mortality. Our meta-analysis of multiple mouse longevity studies also reveals substantial variation in the controls used across experiments, suggesting that a short life span of controls is a potential source of bias. Overall, the present work leads to a reassessment of genes affecting the aging process in mice, with broad implications for our understanding of the genetics of mammalian aging and which genes may be more promising targets for drug discovery.

THE discovery that single-gene manipulations can significantly modulate longevity is arguably the major breakthrough in biogerontology thus far (Kenyon 2010). Genetic manipulations of aging in mice are crucial to gather insights into the underlying mechanisms of aging (de Magalhães and Faragher 2008), to discover pathways modulating longevity (Fernandes *et al.* 2016), and to identify candidate genes for drug discovery (de Magalhães *et al.* 2012; Barardo *et al.* 2017). Moreover, the manipulation of the aging process in mammalian models (particularly mice) via genetic manipulation (gene knockouts, over expression, *etc*.) is crucial to test mechanistic hypotheses of aging (de Magalhães 2005). However, determining if such genetic interventions actually affect the aging process and not some other factor(s) of health is not always straight forward. For example, should a genetic intervention reduce an organism’s resistance to disease, this could conceivably reduce the life span of the organism, although the rate of aging would not have been affected. Differentiating between genetic interventions that affect the life span of an organism through altered health as opposed to changes in the rate of aging is therefore essential to gain insights on aging and determine interventions with wide-ranging effects (Hayflick 2000; de Magalhães *et al.* 2005).

There are two fundamental methods to determine if a life-extending genetic intervention has altered the rate of aging rather than general health. One can track the onset and progression of age-related ailments and physiological degeneration to determine if there is a shift in the onset and progression of the ailments. In addition, efforts have been made to quantify aging rates with mathematical models. The Gompertz law of mortality:

$$R_{m\left(t\right)}=R_{0}e^{\alpha t},$$

where *R_m(t)_* is the hazard or mortality rate (likelihood of death at any time), *t* is age, *R_0_* is the age-independent mortality component, and α is the age-specific exponential coefficient, describes how the hazard rate increases exponentially with age (Finch and Pike 1996). From the Gompertz parameters, the mortality rate doubling time (MRDT) can be calculated. As defined by Finch (1990), the MRDT is the amount of time it takes for the mortality rate to double for a given cohort. A change in MRDT indicates a change in the demographic rate of aging, which is not a perfect reflection of biological aging but a metric that correlates with physiological deterioration and health (Finch 1990; de Magalhães *et al.* 2005). Although some mouse studies have investigated MRDT (Hinkal *et al.* 2009; Lapointe *et al.* 2009), many authors still often assume that changes in the life span of mice following a genetic intervention directly equate to changes in the rate of aging, leading to the misrepresentation of certain genes as having a causal role in aging, when in reality they do not.

Many studies have reported altered median and/or maximum life span as a result of an intervention, but life span alterations may have a number of causes, including altered age at onset of senescence and age-independent mortality (Pletcher *et al.* 2000). To address this lack of distinction, we previously used linear regression to fit the Gompertz model to longevity data from published mouse studies, and statistically compared the rates of aging in these cohorts (de Magalhães *et al.* 2005). For example, we showed that caloric restriction increases the MRDT and thus retards the demographic rate of aging (de Magalhães *et al.* 2005). Here, the same methodology was employed to reassess mouse longevity data published since 2005 and to identify which genes are more important in determining the demographic rate of aging. Lastly, we perform a meta-analysis combining the data from the present study and from our 2005 analysis to investigate patterns in how longevity changes correlate with changes in demographic rates of aging.

## Methods

### Data selection and extraction

Studies published since 2005 were selected since studies published up to 2005 were analyzed previously (de Magalhães *et al.* 2005). Genes were selected from the GenAge database, build 17 (Tacutu *et al.* 2013). GenAge already excludes genes extending life span in short-lived (or disease) mutants or conditions. In addition, some genes were excluded as they could not be studied for demographic parameters (some genes could not be properly fitted to the Gompertz model and some studies lacked full life span data, while other studies lacked sufficient cohort size). A total of 54 genetic manipulations could be properly analyzed (primarily single-gene manipulations but also some manipulations involving more than one gene): 30 genetic manipulations that extended mouse life span (Table 1) and 24 genetic manipulations that reduced life span (Table 2).

**Table 1:** Mortality and life span statistics of genetically altered mouse strains with extended life span

Gene or protein	Type	Strain	Gender	*n*	*t50* (yr)	*tmax* (yr)	Reference
Adcy5	WT	129/SvJ-C57BL/6	M & F	25	2.08	2.75	Yan *et al.* (2007)
	−/−		M & F	13	2.75	3.08	
Agrt1a	WT	C57BL/6 × 129/SvEv	M	10	2.08	2.41	Benigni *et al.* (2009)
	−/−		M	20	2.63	3.00	
Arf/p53	WT	C57BL/6J		111	2.31	3.30	Matheu *et al.* (2007)
	s-Arf/p53			25	2.63	3.03	
Atg5	WT	C57BL/6		65	1.93	2.14	Pyo *et al.* (2013)
	Atg5-Tg			70	2.26	2.52	
BubR1	WT	C57BL/6-SV129	M:30	60	1.72	2.9	Baker *et al.* (2013)
			F: 30				
	mBubR1-Tg		M:29	57	2.02	3.42	
			F:28				
Cat	WT	4033	M & F	44	2.16	2.83	Schriner *et al.* (2005)
	MCAT	4033	M & F	20	2.58	3.10	
	WT	4403	M & F	58	2.18	3.06	
	MCAT	4403	M & F	42	2.60	3.33	
Cisd2	WT (M)	C57BL/6	M	40	2.25	2.90	Wu *et al.* (2012)
	Cisd2-Tg (M)		M	34	2.69	3.07	
	WT (F)		F	25	2.27	2.50	
	Cisd2-Tg (F)		F	21	2.74	3.25	
Clk-1	WT	129Sv/J	F	12	2.01	2.30	Liu *et al.* (2005)
	+/−		F	10	2.34	2.54	
Dgat1	WT	C57BL/6J	F	30	2.04	2.81	Streeper *et al.* (2012)
	−/−		F	30	2.58	3.05	
Esp8	WT	C57BL/6	M:13	29	1.74	2.72	Tocchetti *et al.* (2010)
			F:16				
	−/−		M:20	39	2.16	3.00	
			F:19				
FGF21	WT	C57Bl/6J	M:32	67	2.34	3.56	Zhang *et al.* (2012)
			F:35				
	FGF21-Tg		M:37	77	3.18	NR (> 3.65)	
			F:40				
Ghrh	WT	C57BL/6	M:56	108	1.75	3.20	Sun *et al.* (2013)
		× 129SV	F:52				
	KO		M:39	97	2.55	3.58	
			F:58				
Gpx4	WT	C57BL/6		50	2.63	3.34	Ran *et al.* (2007)
	+/–			50	2.83	3.17	
hMTH1	WT	C57BL/6	M	42	2.17	2.64	De Luca *et al.* (2013)
	hMTH1-Tg		M	34	2.51	3.21	
Htt	WT	129/Sv-	M:1	15	2.34	2.93	Zheng *et al.* (2010)
			F:14				
	ΔQ/ΔQ		M:2	15	2.76	3.43	
			F:13				
Igf1	WT	FVB	M	39	1.97	3.17	Li and Ren (2007)
	Igf1-Tg		M	38	2.43	3.42	
IκB-α	MBH-GFP	C57BL/6		23	2.41	2.91	Zhang *et al.* (2013)
	MBH-IκB-α			31	2.64	3.09	
Irs1	WT (F)	C57BL/6	F	21	2.02	2.86	Selman *et al.* (2009)
	−/− (F)		F	14	2.66	3.61	
Irs2	WT	C57BL/6J	M:21	93	2.36	2.86	Taguchi *et al.* (2007)
			F:30				
	Brain-specific +/−		M:27	65	2.81	3.34	
			F:60				
Mif	WT	C57BL/6J × 129/SvJ	F	24	2.01	2.70	Harper *et al.* (2010)
	−/−		F	39	2.45	3.51	
mTOR	WT	129 × C57BL/6	M & F	34	2.13	3.14	Wu *et al.* (2013)
	KO		M & F	43	2.51	3.14	
Myc	WT (F)	C57BL/6	F	37	2.23	2.86	Hofmann *et al.* (2015)
	+/− (F)		F	39	2.68	3.58	
	WT (M)		M	42	2.41	3.00	
	+/− (M)		M	42	2.66	3.26	
PAPP-A	WT	C57BL6 × 129SV/E	M & F	21	1.84	2.44	Conover and Bale (2007)
	−/−		M & F	20	2.64	3.11	
Pten	WT	C57BL/6	M:49	112	2.17	2.92	Ortega-Molina *et al.* (2012)
		× CBA	F:63				
	Pten-Tg		M:32	64	2.44	3.21	
			F:32				
RpS6K1	WT	C57BL/6	M:26	49	2.23	3.00	Selman *et al.* (2009)
			F:23				
	−/−		M:19	48	2.64	3.40	
			F:29				
RIIβ	WT	C57BL/6 (males)	M	20	2.42	2.79	Enns *et al.* (2009)
	RIIB −/−		M	20	2.75	3.07	
Sirt1	WT	C57BL/6	M & F	31	2.30	3.01	Satoh *et al.* (2013)
	Brain-specific Tg		M & F	34	2.56	3.11	
Slc13a1	WT (M)	C57BL/6J × 129/SV	M	21	1.93	2.60	Markovich *et al.* (2011)
	Nas1 −/− (M)		M	25	2.54	3.30	
	WT (F)		F	34	1.68	2.50	
	Nas1 −/− (F)		F	38	2.06	2.90	
Surf1	WT	BDF1 × cre	M:23	48	1.78	NR	Dell’agnello *et al.* (2007)
			F:25				
	−/−		M:21	43	2.17	2.48	
			F:22				
Tert	Sp53	C57BL/6 × DBA/2	M & F	68	2.13	3.09	Tomás-Loba *et al.* (2008)
	Sp53/TgTert		M & F	56	2.36	3.22	
	Sp53/Sp16/SArf		M & F	39	2.38	3.18	
	Sp53/Sp16/SArf/TgTert		M & F	27	2.38	3.26	

*t50*, median life span; *tmax*, maximum life span; yr, year; WT, wild-type; M, male; F, female; Tg, transgenic; NR, not reported; KO, knockout.

**Table 2:** Mortality and life span statistics of genetically altered mouse strains with shortened life span

Gene or protein	Type	Strain	Gender	*n*	*t50* (yr)	*tmax* (yr)	Reference
Aag, Atm, Mgmt	WT	C57BL/6	M & F	37	2.03	2.87	Meira *et al.* (2014)
	Aag –/–		M & F	29	1.80	2.50	
	Mgmt –/–		M & F	50	1.92	2.77	
	Atm –/–		M & F	19	0.56	1.37	
	Aag –/– Mgmt –/–		M & F	31	1.67	2.71	
ATR	WT		M & F	20	NR	NR	Murga *et al.* (2009)
	ATR^s/s^		M & F	27	0.42	0.65	
Brca1	WT	129O1a × C57BL/6J	F	32	2.15	2.56	Jeng *et al.* (2007)
	+/−		F	26	1.94	2.53	
Bub3 + Rae1	WT	129Sv/E × C57BL/6	N/A	70	2.08	NR	Baker *et al.* (2006)
	+/−, +/−		N/A	100	1.84	NR	
Casp2	WT	C57BL/6	N/A	64	2.62	3.51	Zhang *et al.* (2007)
	−/−		N/A	64	2.62	3.25	
Cdc42	WT	C57BL/6^+/−^ 129/Sv	M & F	16	2.28	2.49	Wang *et al.* (2007)
	−/−		M & F	21	1.09	2.50	
Cisd2	WT	C57BL/6 (B6)	M & F	49	2.09	2.53	Chen *et al.* (2009)
	Cisd2−/−		M & F	16	1.28	2.15	
Cisd2	WT	C57BL/6	M	40	2.25	2.90	Wu *et al.* (2012)
	+/–		M	51	2.05	2.57	
	–/–		M	27	1.76	2.32	
	WT		F	25	2.27	2.50	
	+/–		F	47	1.92	2.50	
	–/–		F	49	1.83	2.94	
DNA pol β	WT	C57BL/6	M	60	2.54	3.19	Cabelof *et al.* (2006)
	+/–		M	67	2.54	3.19	
Fgf-23	−/−	Sv129J	M & F	15	0.12	0.26	Razzaque *et al.* (2006)
Fn1	WT	C57BL/6	M	39	2.43	NR	Muro *et al.* (2003)
	Fn1^EDA −/−^		M	53	1.93	NR	
HtrA2/Omi	mnd2/+;Tg	C57BL/6J	M & F	23	N/A	N/A	Kang *et al.* (2013)
	mnd2/mnd2;Tg		M & F	21	1.28	1.50	
Htr1b	WT		N/A	21	2.58	3.14	Sibille *et al.* (2007)
	−/−		N/A	24	2.05	2.75	
IKK-β	MBH-GFP	C57BL/6	M	23	2.41	2.91	Zhang *et al.* (2013)
	N/*I*kbkb^l/l^		M	24	2.23	2.56	
junD	WT	N/A	M & F	35	1.91	2.41	Laurent *et al.* (2008)
	JunD^−/−^	N/A	M & F	35	1.66	2.17	
Msh2	WT	129S/SvEvTac	M & F	51	NR	NR	Wei *et al.* (2003)
	−/−		M & F	32	0.58	0.92	
Pasg	−/−	129/SvJ/C57BL/6J	N/A	63	0.01	0.07	Sun *et al.* (2004)
Pparg	WT		F	25	2.35	2.68	Argmann *et al.* (2009)
	Pparg2^−/−^		F	26	2.14	2.62	
Sirt7	WT	C57Bl/6 × 129Sv	M	98	NR	NR	Vakhrusheva *et al.* (2008)
	−/−		M	32	0.74	1.60	
Socs2	WT	C57BL/6J × FVB	M & F	123	2.10	3.00	Casellas and Medrano (2008)
	hg/hg		M & F	146	1.33	2.59	
Stub1	WT	C57BL/6 × 129SvEv	M:82	82	2.08	NR	Min *et al.* (2008)
			F:84				
	−/−		M:58	128	0.89	NR	
			F:45				
Trp63	WT	K5CrePR1	NR	74	2.23	3.00	Keyes *et al.* (2005)
	+/−		NR	104	1.74	2.25	
Xrcc5	WT		NR	47	2.04	2.40	Vogel *et al.* (1999)
	Ku86 −/−		NR	89	0.79	1.75	
Xrcc6	WT		M & F	27	2.17	2.80	Li *et al.* (2007)
	Ku70 −/−		M & F	43	0.69	1.45	

*t50*, median life span; *tmax*, maximum life span; yr, year; WT, wild-type; M, male; F, female; Tg, transgenic; NR, not reported;

Mortality data were extracted from published studies. WebPlotDigitizer, an online graph digitizer application (https://automeris.io/WebPlotDigitizer/), was used to extract raw data from survival graphs in some cases. The digital imaging software package PaintShop Pro X3 (Corel Corporation, Ottawa, Canada) was used to extract age-specific survival data from published survivorship graphs (usually in the form of Kaplan–Meier survivorship curves). Percent survival was extracted at regular time intervals over the linear phase of these plots. Interval length was selected for each study individually to maximize the number of consecutive time points analyzed within this exponential phase.

### Demographic analysis

The same methods and computer programs for the data gathering and analysis of de Magalhães *et al.* (2005) were used. Once the mortality data were collected, the age-specific mortality (*q_x_*) was calculated as the number of mice alive (*T*_1_) at the beginning of a given time interval minus the number of mice alive (*T*_2_) at the end of that same time interval divided by *T*_1_ (*i.e.*, (T1−T2)/T1 = qx). The hazard rate (*hz*) for each individual time interval was calculated as *hz* = (T1−T2)/((T1+T2)/2) or the number of animals dying in the interval divided by the average number of individuals alive in the interval. The aging rate was then calculated through use of the Gompertz equation: *R_m(t)_ = R_0_e*^α^*^t^*; where *R_m(t)_* is the chance of dying (the hazard rate) at age *t*, *R_0_* is the nonexponential factor in mortality, and α is the exponential parameter (Finch *et al.* 1990). The Gompertz model was used because, as de Magalhães *et al.* (2005) asserted, the sample size (number of mice) in the majority of these experiments was small, meaning that other logarithmic methods of fitting models to mortality data may not provide the accuracy that the Gompertz model gives while retaining its simplicity. This point held true for the majority of genetic interventions tested herein and so it was decided that use of the Gompertz model remained a viable option for this analysis. From the Gompertz model, the weighted regression line (weighted by the number of animals dying at each interval) was calculated by *ln(R_m(t)_) = ln(R_0_) +* α*t*, which will also give the MRDT as 0.693/α. To compare α between a given genetic intervention cohort and wild-type (WT) mice from the same lineage, a “dummy variables” test was employed as described (de Magalhães *et al.* 2005). As in de Magalhães *et al.* (2005), the aim of this research was not to find the best fit model to describe the whole of the mortality curves, but rather to find if any previously published genetic interventions have a statistically significant effect on the exponential increase in mortality (α) and hence on the demographic rate of aging. Consequently, the simpler, nested Gompertz model was preferred (de Magalhães *et al.* 2005). Besides, data were only analyzed from the onset of the exponential increase in mortality, though typically < 10% of animals were left out.

### Statistical analysis

A “dummy variables” method was used to compare the slopes of the hazard functions obtained through linear regression for the WT and test cohorts and a two-tailed Student’s *t*-test was applied to evaluate whether they were significantly different (*P* < 0.05). Analysis was performed in SPSS version 22 (IBM) using our previous scripts (de Magalhães *et al.* 2005) (code available in the supplemental material and at http://genomics.senescence.info/software/demographic.html).

### Data availability

The survival data used in this study is provided in the Supplemental Material, Tables S1 and S2 in File S1. The SPSS code used is provided in File S2.

## Results

The Gompertz law of mortality describes the exponential increase in mortality rate with age. Modified versions of this function exist to model mortality deceleration observed at very young and very old ages; however, the simplest form was chosen to fit the mouse survivorship data in this study because the mouse cohorts are often small (*n* < 50). The basic Gompertz model is described by only two parameters, so is more suitable for fitting data from smaller sample sizes (Pletcher 2002) and increases the ease of comparing aging rates between cohorts. Since mouse longevity data are largely presented in publications as Kaplan–Meier survival curves, the life spans were divided into discrete time intervals and then linear regression was used to calculate age-specific mortality rates for each interval (see *Methods*). This allowed an estimation of Gompertz parameters for each cohort that could be directly compared.

In this study, 54 previously published genetic manipulations that have been associated with alterations in mouse life span were analyzed; 30 manipulations previously reported as having a life span-extending effect (Table 1) and 24 that were previously reported as having a life span-reducing effect (Table 2). The aim of this study was to reassess genes that have been reported to regulate longevity in mice to ascertain which of them might exert this effect through regulating the rate of aging.

### Analysis of life-extending gene manipulations

Of the 30 genetic manipulations previously reported as having life span-increasing effects, we found 13 genes to have a statistically significant effect on the demographic rate of aging (Table 3): *BubR1*, *Cisd2*, *Dgat1*, *Fgf21*, *Ghrh*, *Gpx4*, *hMTH1*, *Irs2*, *mTOR*, *Sirt1*, *Slc13a1*, *Surf1*, and *Tert*. However, surprisingly only two of these genes (*Cisd2* and *hMTH1*) retarded the demographic rate of aging. Full survival data are provided in the supplemental material (Table S1 in File S1).

**Table 3:** Gompertz parameters for genetically altered mouse strains with extended life span from a regression line calculated by *ln(R_m(t)_) = ln(R_0_) +* α*t* (see *Methods*)

Gene or protein	Type	Strain	α	α SE	ln(R_0_)	*r*^2^	MRDT (yr)
Adcy5	WT	129/SvJ-C57BL/6	3.87	0.94	−9.61	0.71	0.18
	−/−		5.95	0.34	−17.20	0.97	0.12
Agrt1a	WT	C57BL/6 × 129/SvEv	5.20	1.21	−11.15	0.86	0.13
	−/−		3.95	1.30	−10.81	0.70	0.18
Arf/p53	WT	C57BL/6J	2.88	0.32	−7.59	0.94	0.24
	s-Arf/p53		2.80	0.44	−8.05	0.86	0.25
Atg5	WT	C57BL/6	7.30	5.48	−14.40	0.47	0.10
	Atg5-Tg		8.92	1.31	−21.54	0.82	0.08
BubR1*^a^*	WT	C57BL/6-SV129	1.62	0.16	−5.42	0.92	0.43
	mBubR1-Tg		2.61	0.097	−7.48	0.93	0.27
Cat	WT	4033	2.30	0.18	−6.40	0.81	0.30
	MCAT	C57BL/6J	3.00	0.59	−8.80	0.68	0.23
	WT	4403	2.00	0.21	−5.93	0.62	0.35
	MCAT		2.20	0.20	−7.41	0.78	0.32
Cisd2*^a^*	WT male	C57BL/6	7.67	2.86	−17.79	0.71	0.09
	Cisd2-Tg male		6.96	1.84	−19.39	0.83	0.10
	WT female		6.62	2.27	−14.43	0.90	0.10
	Cisd2-Tg female		2.40	0.59	−7.10	0.74	0.29
Clk-1	WT	129Sv/J	3.09	0.64	−6.05	0.89	0.22
	+/−		2.49	0.92	−5.95	0.64	0.28
Dgat1*^a^*	WT	C57BL/6J	1.92	0.53	−5.12	0.66	0.36
	−/−		3.56	0.20	−9.63	0.99	0.19
Esp8	WT	C57BL/6	1.50	0.22	−3.70	0.73	0.46
	−/−		1.98	0.18	−5.15	0.81	0.35
FGF21*^a^*	WT	C57Bl/6J	1.82	0.35	−5.66	0.82	0.38
	FGF21-Tg		2.04	0.40	−7.89	0.87	0.34
Ghrh*^a^*	WT	C57BL/6 × 129SV	1.31	0.18	−3.55	0.90	0.53
	KO		1.53	0.17	−5.12	0.94	0.45
Gpx4*^a^*	WT	C57BL/6	3.11	1.08	−9.18	0.73	0.22
	+/–		5.43	0.72	−15.77	0.95	0.13
hMTH1*^a^*	WT	C57BL/6	3.50	0.65	−8.78	0.81	0.20
	hMTH1-Tg		2.29	0.47	−6.91	0.72	0.30
Htt	WT	129/Sv-	2.30	1.01	−6.03	0.56	0.30
	ΔQ /ΔQ		1.80	0.51	−5.19	0.81	0.39
Igf1	WT	FVB	1.06	0.26	−3.02	0.74	0.65
	Igf1		1.35	0.25	−4.43	0.77	0.51
IκB-α	MBH-GFP	C57BL/6	4.15	0.74	−10.75	0.65	0.17
	MBH-DN IB-α		4.43	0.76	−12.86	0.62	0.16
Irs1	WT (female)	C57BL/6	2.18	0.26	−5.00	0.80	0.32
	−/− (female)		1.94	0.31	−6.45	0.71	0.36
Irs2*^a^*	WT	C57BL/6J	2.99	0.13	−7.29	0.87	0.23
	Brain-specific +/−		6.37	0.33	−17.73	0.85	0.11
Mif	WT	C57BL/6J × 129/SvJ	2.45	0.40	−6.08	0.84	0.28
	−/−		1.90	0.31	−6.02	0.82	0.37
mTOR*^a^*	WT	129 × C57BL/6	1.47	0.45	−4.46	0.68	0.47
	KO		2.41	0.15	−6.94	0.98	0.29
Myc	WT female	C57BL/6	2.19	0.55	−5.88	0.76	0.32
	+/– female		3.19	1.05	−9.64	0.70	0.22
	WT male		2.53	0.21	−7.06	0.96	0.27
	+/– male		2.55	0.36	−7.75	0.90	0.27
PAPP-A	WT	C57BL6 × 129SV/E	2.33	0.35	−5.28	0.71	0.30
	−/−		2.67	0.32	−7.39	0.80	0.26
Pten	WT	C57BL/6 × CBA	2.33	0.14	−5.83	0.97	0.30
	Pten-Tg		2.52	0.24	−7.07	0.95	0.27
RpS6K1	WT	C57BL/6	1.81	0.069	−4.64	0.93	0.38
	−/−		1.73	0.056	−5.30	0.91	0.40
RIIβ	WT	C57BL/6 (males)	3.00	0.31	−7.68	0.85	0.23
	RIIB −/−		3.55	0.34	−10.09	0.88	0.20
Sirt1*^a^*	WT	C57BL/6	2.45	0.49	−7.20	0.81	0.28
	Brain-specific Tg		3.63	0.63	−10.71	0.83	0.19
Slc13a1*^a^*	WT (male)	C57BL/6J × 129/SV	1.79	0.37	−4.34	0.83	0.39
	Nas1 −/− (male)		1.70	0.47	−5.02	0.72	0.41
	WT (female)		0.87	0.13	−2.95	0.86	0.80
	Nas1 −/− (female)		1.69	0.29	−4.31	0.85	0.41
Surf1*^a^*	WT	BDF1 × cre	2.19	0.39	−4.81	0.86	0.32
	−/−		3.41	0.47	−7.82	0.96	0.20
TgTert*^a^*	Sp53	C57BL/6 × DBA/2	2.74	0.33	−6.51	0.95	0.25
	Sp53*^a^* /TgTert		3.34	0.41	−8.14	0.94	0.21
	Sp53/Sp16/SArf		1.47	0.58	−4.86	0.52	0.47
	Sp53/Sp16/SArf/TgTert		2.08	0.65	−7.24	0.77	0.33

α, age-specific exponential coefficient; ln(R0), nonexponential factor in mortality; MRDT, mortality rate doubling time as in 0.693/α; yr, year; WT, wild-type; Tg, transgenic; KO, knockout.

a Indicates genes for which the changes in MRDT were statistically significant (*P* < 0.05).

**Figure 1:**
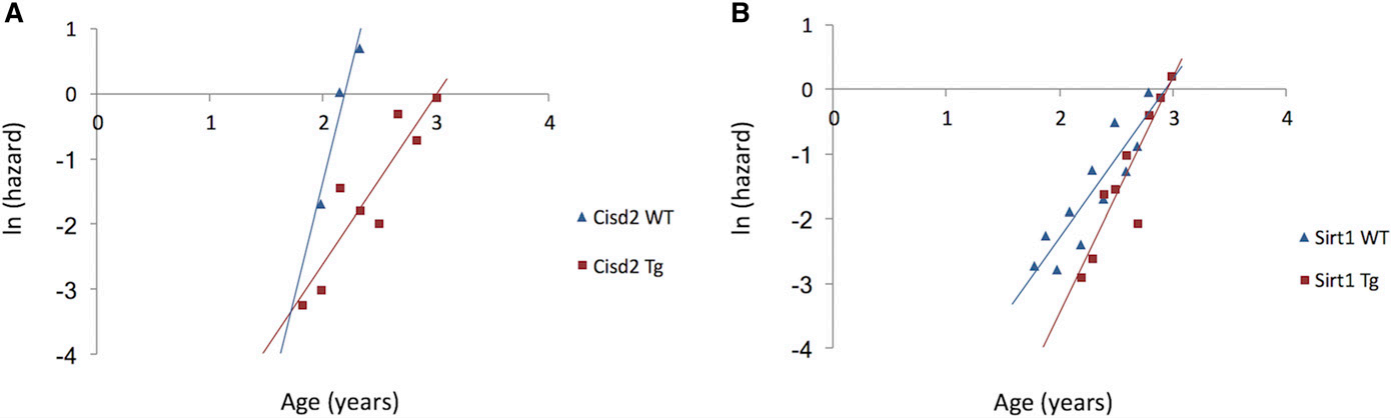
Natural logarithm of mortality rates for wild-type (WT, blue diamonds) and experimental transgenic (Tg, red squares) cohorts. Lines represent estimated adult mortality trajectories based on Gompertz parameters for the WT (blue lines) and experimental (red lines) cohorts. (A) *Cisd2*. (B) *Sirt1*.

A few notable examples are worth emphasizing. Transgenic expression of *Cisd2* in female mice produced persistent expression of the Cisd2 protein in contrast to levels in WT mice, which diminished with age (Wu *et al.* 2012). Our analysis showed that this resulted in a nearly twofold higher MRDT compared to that of female WT controls, consistent with a difference between the Gompertz curve gradients (Figure 1A). This suggests that *Cisd2* regulates the demographic rate of aging. However, the nature of the role of *Cisd2* in aging is confused by the fact that reduced expression of *Cisd2* in heterozygous and double-knockout female mice from the same study also resulted in a significantly increased MRDT (see Table 4), suggesting that reduced *Cisd2* expression also slowed the demographic rate of aging.

**Table 4:** Gompertz parameters for genetically altered mouse strains with shortened life span from a regression line calculated by *ln(R_m(t)_) = ln(R_0_) +* α*t* (see *Methods*)

Gene or protein	Type	Strain	α	α SE	ln(R_0_)	*r*^2^	MRDT (yr)
Aag, Atm, Mgmt*^a^*	WT	C57BL/6	3.81	0.82	−8.83	0.78	0.18
	Aag –/–*^a^*		2.20	1.04	−5.38	0.53	0.31
	Mgmt –/–		3.08	0.50	−7.65	0.86	0.23
	Atm –/–*^a^*		2.21	1.03	−2.39	0.48	0.31
	Aag –/– Mgmt –/–*^a^*		1.66	0.41	−4.38	0.65	0.42
ATR	WT	N/A	N/A	N/A	N/A	N/A	N/A
	ATR^s/s^		6.46	1.33	−3.38	0.86	0.11
Brca1	WT	129O1a × C57BL/6J	4.59	0.36	−10.01	0.98	0.15
	+/− (female)		3.96	0.48	−8.06	0.97	0.17
Bub3 + Rae1	WT	129Sv/E × C57BL/6	1.54	0.34	−4.86	0.91	0.45
	+/−, +/−		1.16	0.49	−3.72	0.61	0.60
Casp2*^a^*	WT	C57BL/6	1.46	0.31	−5.39	0.79	0.47
	−/−		3.20	0.44	−9.44	0.91	0.22
Cd42 GAP*^a^*	WT	C57BL/6^+/−^ 129/Sv	3.39	1.11	−8.16	0.61	0.20
	−/−		1.24	1.44	−2.89	0.87	0.56
Cisd2*^a^*	WT	C57BL/6 (B6)	3.42	0.81	−8.37	0.64	0.20
	Cisd2−/−		1.02	0.36	−2.10	0.66	0.68
Cisd2*^a^*	WT male	C57BL/6	7.67	2.86	−17.79	0.71	0.09
	+/– male*^a^*		4.06	1.26	−9.68	0.68	0.17
	–/– male		5.56	1.11	−11.06	0.86	0.12
	WT female		6.62	2.27	−14.43	0.90	0.10
	+/– female*^a^*		2.50	0.49	−5.69	0.81	0.28
	–/– female*^a^*		2.44	0.40	−5.16	0.84	0.28
DNA pol β	WT	C57BL/6	3.06	0.59	−9.42	0.79	0.23
	+/–		3.11	0.65	−9.11	0.82	0.22
Fgf-23	−/−	Sv129J	6.48	N/A	−1.76	0.41	0.11
Fn1*^a^*	WT	C57BL/6	1.49	0.48	−5.22	0.62	0.46
	Fn1^EDA −/−^		2.73	0.56	−6.01	0.82	0.25
Htr1b*^a^*	WT		4.95	0.83	−13.74	0.90	0.14
	−/−		4.06	0.46	−9.56	0.94	0.17
HtrA2/Omi	mnd2/mnd2;Tg	C57BL/6J	N/A	N/A	N/A	N/A	N/A
	mnd2/+;Tg		5.38	1.69	−7.16	0.84	0.13
IKK-β*^a^*	MBH-GFP	C57BL/6	4.78	1.09	−12.43	0.76	0.15
	N/*I*kbkb^l/l^		6.03	0.84	−14.37	0.90	0.12
junD*^a^*	WT	N/A	2.67	0.77	−5.85	0.75	0.26
	JunD^−/−^	N/A	3.89	0.57	−7.47	0.90	0.18
Msh2	WT	129S/SvEvTac	N/A	N/A	N/A	N/A	N/A
	−/−		1.69	N/A	−2.69	0.26	0.41
Pasg	−/−	129/SvJ/C57BL/6J	64.15	N/A	−2.55	0.75	0.01
Pparg*^a^*	WT		4.43	0.53	−11.02	0.93	0.16
	Pparg2^−/−^		2.59	0.29	−6.93	0.92	0.27
Sirt7	WT	C57Bl/6 × 129Sv	N/A	N/A	N/A	N/A	N/A
	−/−		2.16		−2.01	0.71	0.32
Socs2*^a^*	WT	C57BL/6J × FVB	2.14	0.40	−5.16	0.85	0.32
	hg/hg		1.43	0.33	−2.69	0.73	0.48
Stub1*^a^*	WT	C57BL/6 × 129SvEv	0.80	0.40	−3.78	0.44	0.87
	−/−		1.31	0.44	−2.46	0.59	0.53
Trp63*^a^*	WT	K5CrePR1	2.90	N/A	−7.47	0.86	0.24
	+/−		1.75	N/A	−3.72	0.83	0.40
Xrcc5*^a^*	WT		2.68	0.35	−5.84	0.94	0.26
	Ku86 −/−		1.63	0.38	−2.34	0.70	0.43
Xrcc6*^a^*	WT		1.92	0.30	−4.98	0.89	0.36
	Ku70 −/−		1.39	0.44	−1.49	0.83	0.50

α, age-specific exponential coefficient; ln(R0), nonexponential factor in mortality; MRDT, mortality rate doubling time as in 0.693/α; yr, year; WT, wild-type; N/A, not applicable; Tg, transgenic.

a Indicates genes for which the changes in MRDT were statistically significant (*P* < 0.05).

Most genes examined did not impact on MRDT, and a few even reduced the MRDT. Of note, regarding *Sirt1*, a 33% reduction in MRDT was observed for transgenic mice with brain-specific overexpression of this gene. This is supported by a visible change in the slope of the Gompertz curve (Figure 1B). It therefore appears that the increases in median and maximum life span reported (Satoh *et al.* 2013) are not mediated by a decrease in the rate of aging. Instead, it appears that the longevity extension in this cohort occurred through delayed onset of the exponential increase in mortality rate. Likewise, mice constitutively overexpressing both the tumor suppressor *p53* and telomerase reverse transcriptase (*Tert*) exhibited enhanced 3-year survival rates compared to super-p53 mice expressing only the additional transgenic copy of *p53* (Tomás-Loba *et al.* 2008). Interestingly, our analysis indicates that the MRDT of the former cohort was modestly reduced by 18% compared to that of the control mice, which suggests that the life-extending effect of *Tert* overexpression is due to a reduced *R_0_* rather than to a slower demographic rate of aging. By contrast, mice overexpressing *Tert* in addition to *p16*, *Arf*, and *p53* have a higher MRDT, although this difference was not statistically significant (Table 3).

### Analysis of genes reported to reduce life span and/or accelerate aging

Interventions that reduce life span by increasing the rate of aging would be expected to reduce the MRDT. Of the 24 genes previously reported as having life span-reducing effects, we found 15 to have a statistically significant effect on the demographic rate of aging (Table 4): *Aag/Atm/Mgmt*, *Casp2*, *Cisd2* (2× studies), *Cdc42GAP*, *Fn1*, *Htr1b*, *IKK*-β, *JunD*, *Pparg*, *Socs2*, *Stub1*, *Trp63*, *Xrcc5*, and *Xrcc6*. Five of these (*Casp2*, *Fn1*, *IKK*-β, *JunD*, and *Stub1*) accelerated demographic aging. Full survival data are provided in the supplemental material (Table S2 in File S1).

**Figure 2:**
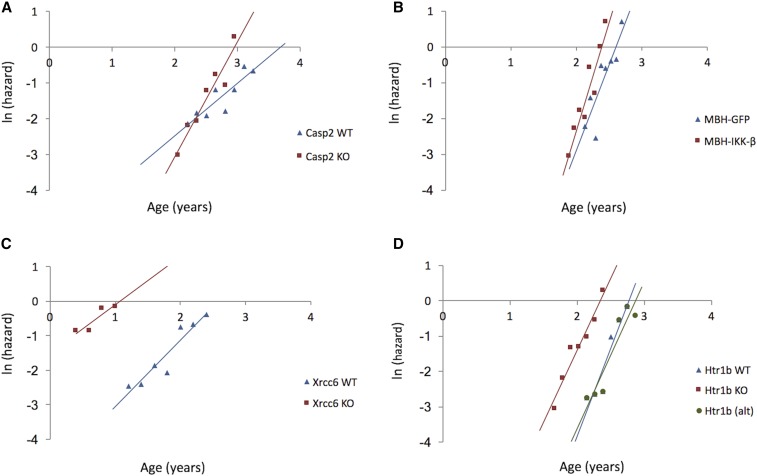
Natural logarithm of mortality rates for wild-type (WT, blue diamonds) and experimental (red squares) cohorts. Lines represent estimated adult mortality trajectories based on Gompertz parameters for the WT (blue lines) and experimental (red lines) cohorts. KO, knockout. (A) *Casp2*; (B) *MBH-IKK*-β; (C) *Xrcc6*; and (D) *Htr1b* with the green line and circles representing an alternative for the WT cohort (see text for details).

As before, a few notable examples are worth highlighting. Mice carrying a double knockout of *Casp2* exhibited a 54% lower MRDT than WT littermates (Figure 2A). Therefore, as initially reported by Zhang *et al.* (2007), *Casp2* accelerates the rate of aging in mice. Zhang *et al.* (2007) used several methods including a thorough detailing of the progression of age-related ailments (such as gradual hair and increased bone loss) to detail the rate of aging of the *Casp2* knockout and their WT littermates, and our new research (using statistical demographic methods) has drawn the same conclusions.

Another gene manipulation that significantly reduced MRDT involved IκB kinase-β (IKK-β), which is involved in the activation of NF-κB. NF-κB activity in the hypothalamus of mice increases with age and mice expressing constitutively active IKK-β in the mediobasal hypothalamus (MBH) exhibited shortened life spans (Zhang *et al.* 2013). The MRDT for MBH-IKK-β mice was reduced by 21% compared to that of control mice. The fitted Gompertz curves showed an increased gradient (Figure 2B), providing evidence that activated hypothalamic NF-κB promotes faster aging. As the authors note, this is interesting because it suggests that a single organ, the hypothalamus, is important in regulating aging of the whole animal.

As in life-extending interventions, most genes did not impact on MRDT and some even had an opposite effect than expected. For example, *Xrcc6* −/− (also known as *Ku70* −/−) mice had shortened life spans compared to WT controls (Li *et al.* 2007). Fitting the survival data from this study produced mortality curves with visibly different starting ages (Figure 2C) and the MRDT of the *Xrcc6* −/− mice was 38% larger than that of the WT cohort. This points to a slower demographic rate of aging in the *Xrcc6* −/− mice. It should be noted that animals that died in the first 3 weeks were censored in the original study because *Ku* mutant mice frequently do not survive to weaning age. Including these animals would have altered the survival plots and might therefore have impacted on the outcome of this analysis.

Finally, reassessment of the survival data of *Htr1b*−/− mice showed that they have a 22% increased MRDT compared to WT controls. This can be seen as a slight difference in the slopes of the Gompertz curves (Figure 2D). Reduced longevity was reported in *Htr1b*−/− mice (Sibille *et al.* 2007) and our results support the conclusion that *Htr1b* deficiency produces a significantly decreased demographic rate of aging; however, the cohorts used in this study were small (*n* = 21 for WT and *n* = 24 for *Htr1b*−/−). If we increase the period from which data were extracted for the *Htr1b*−/− mice and exclude outliers of the Gompertz curve, the difference in MRDT is no longer significant. This result highlights the differences that one can obtain in this type of analysis by changing subjective parameters, in particular for smaller cohorts.

### Longevity effects are driven by aging-independent mortality

Looking at our data set as a whole (Table 1, Table 2, Table 3, and Table 4), it is clear that studies are highly variable. Of note, cohort size ranges from 10 to 146 animals. Moreover, while the SD of median life span (*t50*) was only 13% for life span-extending manipulations (range 1.68–3.18 years), for life span-reducing manipulations it was 39% (0.01–2.62 years range). For maximum life span (*tmax*), SD was 11% for life span-extending manipulations (range 2.14–3.61 years) and 33% (0.07–3.51 years range) for life span-reducing manipulations. This is not surprising given that life-shortening manipulations can have greater effect sizes than life-extending manipulations, but it also introduces noise in demographic aging estimates, in particular for life span-shortening manipulations.

Even looking only at WT controls from the C57BL/6 strain (15 studies), the most common strain in our analysis, the range of *t50* was 1.74–2.63 years while *tmax* ranged from 2.14 to 3.56. While *tmax* is influenced by cohort size, *t50* is not and, therefore, this substantial variation for WT mice of the same genetic background suggests that considerable variation is introduced by differences in animal husbandry and stochastic factors. Relative SD for α and ln(*R_0_*) in C57BL/6 cohorts were, respectively, 57 and 42%.

We also investigated if, in life span-extending manipulations, there is a negative correlation between the *t50* of the controls and the life extension effects (measured as the percentage *t50* increase in the experimental cohort). Indeed, there is a moderate (*r^2^* = 0.30) but statistically significant negative correlation (*P*-value = 0.002; *n* = 33), suggesting that effect sizes in longevity experiments could be influenced by the short life span of the controls.

There was a strong negative correlation between α and ln(*R_0_*): *r^2^* = 0.93 for life span-extending manipulations and *r^2^* = 0.53 (after removing *Pasg*, which is an outlier; see Table 4) for life span-reducing manipulations. As such, increases in aging-independent mortality tend to be accompanied by a slower demographic aging rate, as observed before (de Magalhães *et al.* 2005).

**Figure 3:**
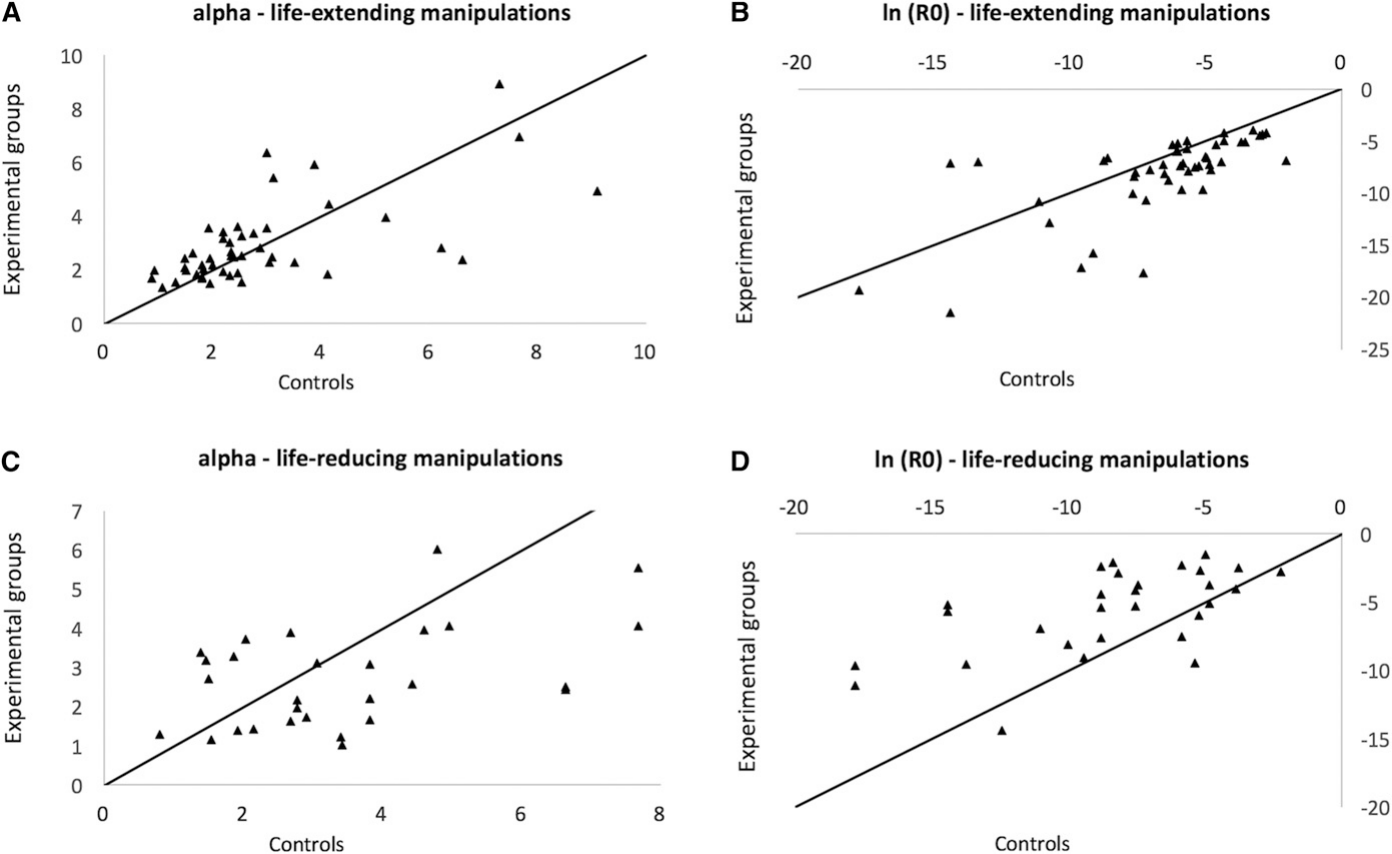
Comparison between control (*x*-axis) and experimental cohorts (*y*-axis) for α in life-extending manipulations (A), *R_0_* in life-extending manipulations (B), α in life span-reducing manipulations (C), and *R_0_* in life span-reducing manipulations (D).

Combining earlier results (de Magalhães *et al.* 2005) with the current analysis allows greater power to evaluate the usefulness of demographic analysis in aging. Therefore, we employed a data set with 63 manipulations of longevity: 41 life span-extending plus 22 life span-reducing manipulations (note that for this analysis we excluded manipulations for which we lacked demographic aging parameters for controls, which resulted in the exclusion of six life-reducing interventions from the previous analyses). Of note, we found that by and large an impact on longevity is caused by a change in the aging-independent mortality, which is observed both for life-extending (Figure 3, A and B) and life-reducing manipulations (Figure 3, C and D). And confirming the above-mentioned results, we observed that decreases in ln(*R_0_*) correlated with increases in α for life span-extending (*r^2^* = 0.64) and life span-reducing manipulations (*r^2^* = 0.64).

## Discussion

The Gompertz function, used here to fit only the linear phase of the mortality trajectories, allowed for simple comparison between studies as it is described by just two parameters. Although it has been found that more complex adaptations of the Gompertz function [*e.g.*, the logistic model (Pletcher *et al.* 2000)] provide a better fit for some whole-mortality curves (de Magalhães *et al.* 2005; Yen *et al.* 2008), the sample sizes usually reported for mouse life span experiments are insufficiently large to apply these models with sufficient accuracy.

Longevity is influenced by a number of factors, including age-independent mortality, age at the onset of senescence, and demographic rate of aging. In this study, we employed the Gompertz model to fit published mouse survival data and generate parameters that could be used to identify genes that influence the demographic rate of aging. Overall, only 7/54 genes were found to have a statistically significant effect on the demographic rate of aging as expected from longevity manipulations. These results suggest that only a relatively small proportion of interventions reported to affect longevity in mice do so through directly influencing the demographic rate of aging, in line with other, albeit smaller, studies (de Magalhães *et al.* 2005; Yen *et al.* 2008; Garratt *et al.* 2016; Hughes and Hekimi 2016). Surprisingly, 20/54 genes had a statistically significant impact on the demographic rate of aging in the opposite direction than would be expected for the published longevity effects. One possible explanation is that many mutations impacted on various parameters affecting longevity in nonlinear ways, and indeed we observed that increases in aging-independent mortality correlated with a slower demographic aging rate. For instance, *Sirt1* deficiency extended life span but increased the demographic rate of aging; its effect appeared to be exerted instead by delaying the age of onset of mortality rate escalation. This highlights the complex relationship between life span and the demographic rate of aging. It is also possible that other confounding factors, like censored data or noise, in particular for smaller cohorts, influenced these results.

Another caveat of our approach concerns the number of mice used in some of the original studies, which ranged from 10 to 146 animals per cohort. While research reported here has attempted to compensate for this by using the Gompertz equation, which allows for small sample sizes, one cannot escape the low statistical power that accompanies such small sample sizes. Interestingly, caloric restriction has been shown to significantly retard the demographic rate of aging, but this was a large study with > 200 animals in total (de Magalhães *et al.* 2005). Therefore, caution must be taken when interpreting some of the results detailed here from studies with small sample sizes. Indeed, we observed that, in smaller experimental cohorts, subjective decisions in estimating Gompertz parameters can significantly affect the results, *e.g.*, for *Htr1b*−/−.

Potential caveats of our analysis include the subjectivity of deciding the time at which analysis should begin and problems in the reporting of mouse survival data. As reviewed in detail by Ladiges *et al.* (2009), mouse longevity studies should adhere to certain standards to provide useful data. These include a sufficiently large cohort, high standards of pathogen-free animal husbandry to eliminate deaths from infectious disease, and separate reporting of male and female survival data. Unfortunately, life span data are often incompletely reported and, in many of the studies analyzed in this work, male and female data were not presented separately. Censoring of mice that died before a certain age in some studies may also have introduced noise into our results.

Our results provide insights regarding the role in aging of various genes. Of particular interest are the findings concerning *Casp2* and *Cisd2*. We found that *Casp2* deficiency increased the demographic rate of aging, which has not previously been proposed. *Casp2* −/− mice had a similar median life span to WT and did not show elevated tumor incidence (Zhang *et al.* 2007). Besides, our results indicate that persistent expression of *Cisd2* significantly reduced the demographic rate of aging. Further investigation has since strengthened the case for Cisd2's involvement in regulating the rate of aging by showing that it is involved in autophagy, mitochondrial function, and adipocyte differentiation (Wang *et al.* 2014), showing that it may influence several pathways thought to be important for aging.

### Conclusions

Overall, we performed a demographic analysis of 54 mouse studies in which genetic manipulations significantly extended or reduced life span. We also combined our results with a previous report to perform an analysis of factors associated with longevity in mice. To our knowledge, this is the largest such study to date. Our main conclusions are: (1) most genetic manipulations of longevity in mice do so by modulating aging-independent mortality; (2) there is substantial variation in the life span of controls of the same strain across experiments; (3) studies in which the life span of the controls is short have a greater life span increase, emphasizing the importance of having adequate control groups; (4) mouse life span studies employing small cohorts can yield unreliable results; (5) life span-reducing experiments tend to be noisier and more difficult to analyze for demographic parameters than life-extending experiments; and (6) a greater aging-independent mortality is usually accompanied by a slower demographic aging rate.

## Supplementary Material

Supplemental material is available online at www.genetics.org/lookup/suppl/doi:10.1534/genetics.118.300821/-/DC1.

## References

[bib1] Argmann C. Dobrin R. Heikkinen S. Auburtin A. Pouilly L., 2009 Ppargamma2 is a key driver of longevity in the mouse. PLoS Genet. 5: e1000752 10.1371/journal.pgen.1000752 19997628 PMC2780700

[bib2] Baker D. J. Jeganathan K. B. Malureanu L. Perez-Terzic C. Terzic A., 2006 Early aging-associated phenotypes in Bub3/Rae1 haploinsufficient mice. J. Cell Biol. 172: 529–540. 10.1083/jcb.200507081 16476774 PMC2063673

[bib3] Baker D. J. Dawlaty M. M. Wijshake T. Jeganathan K. B. Malureanu L., 2013 Increased expression of BubR1 protects against aneuploidy and cancer and extends healthy lifespan. Nat. Cell Biol. 15: 96–102. 10.1038/ncb2643 23242215 PMC3707109

[bib4] Barardo D. Thornton D. Thoppil H. Walsh M. Sharifi S., 2017 The DrugAge database of aging-related drugs. Aging Cell 16: 594–597. 10.1111/acel.12585 28299908 PMC5418190

[bib5] Benigni A. Corna D. Zoja C. Sonzogni A. Latini R., 2009 Disruption of the Ang II type 1 receptor promotes longevity in mice. J. Clin. Invest. 119: 524–530. 10.1172/JCI36703 19197138 PMC2648681

[bib6] Cabelof D. C. Ikeno Y. Nyska A. Busuttil R. A. Anyangwe N., 2006 Haploinsufficiency in DNA polymerase beta increases cancer risk with age and alters mortality rate. Cancer Res. 66: 7460–7465. 10.1158/0008-5472.CAN-06-1177 16885342

[bib7] Casellas J. Medrano J. F., 2008 Lack of Socs2 expression reduces lifespan in high-growth mice. Age (Dordr.) 30: 245–249. 10.1007/s11357-008-9064-1 19424848 PMC2585654

[bib8] Chen Y. F. Kao C. H. Chen Y. T. Wang C. H. Wu C. Y., 2009 Cisd2 deficiency drives premature aging and causes mitochondria-mediated defects in mice. Genes Dev. 23: 1183–1194. 10.1101/gad.1779509 19451219 PMC2685531

[bib9] Conover C. A. Bale L. K., 2007 Loss of pregnancy-associated plasma protein A extends lifespan in mice. Aging Cell 6: 727–729. 10.1111/j.1474-9726.2007.00328.x 17681037

[bib10] Dell’agnello C. Leo S. Agostino A. Szabadkai G. Tiveron C., 2007 Increased longevity and refractoriness to Ca(2+)-dependent neurodegeneration in Surf1 knockout mice. Hum. Mol. Genet. 16: 431–444. 10.1093/hmg/ddl477 17210671

[bib11] De Luca G. Ventura I. Sanghez V. Russo M. T. Ajmone-Cat M. A., 2013 Prolonged lifespan with enhanced exploratory behavior in mice overexpressing the oxidized nucleoside triphosphatase hMTH1. Aging Cell 12: 695–705. 10.1111/acel.12094 23648059

[bib12] de Magalhães J. P., 2005 Open-minded scepticism: inferring the causal mechanisms of human ageing from genetic perturbations. Ageing Res. Rev. 4: 1–22. 10.1016/j.arr.2004.05.003 15619467

[bib13] de Magalhães J. P. Faragher R. G., 2008 Cell divisions and mammalian aging: integrative biology insights from genes that regulate longevity. BioEssays 30: 567–578. 10.1002/bies.20760 18478536

[bib14] de Magalhães J. P. Cabral J. A. Magalhaes D., 2005 The influence of genes on the aging process of mice: a statistical assessment of the genetics of aging. Genetics 169: 265–274. 10.1534/genetics.104.032292 15466429 PMC1448866

[bib15] de Magalhães J. P. Wuttke D. Wood S. H. Plank M. Vora C., 2012 Genome-environment interactions that modulate aging: powerful targets for drug discovery. Pharmacol. Rev. 64: 88–101. 10.1124/pr.110.004499 22090473 PMC3250080

[bib16] Enns L. C. Morton J. F. Treuting P. R. Emond M. J. Wolf N. S., 2009 Disruption of protein kinase A in mice enhances healthy aging. PLoS One 4: e5963 (erratum: PLoS One 5: DOI: 10.1371/annotation/c7cad2dc-1eca-487e-89ae-151a22d8a0b4). 10.1371/journal.pone.0005963 19536287 PMC2693670

[bib17] Fernandes M. Wan C. Tacutu R. Barardo D. Rajput A., 2016 Systematic analysis of the gerontome reveals links between aging and age-related diseases. Hum. Mol. Genet. 25: 4804–4818. 28175300 10.1093/hmg/ddw307 PMC5418736

[bib18] Finch C. E., 1990 *Longevity*, *Senescence*, *and the Genome*. The University of Chicago Press, Chicago.

[bib19] Finch C. E. Pike M. C., 1996 Maximum life span predictions from the Gompertz mortality model. J. Gerontol. A Biol. Sci. Med. Sci. 51: B183–B194. 10.1093/gerona/51A.3.B183 8630694

[bib20] Finch C. E. Pike M. C. Witten M., 1990 Slow mortality rate accelerations during aging in some animals approximate that of humans. Science 249: 902–905. 10.1126/science.2392680 2392680

[bib21] Garratt M. Nakagawa S. Simons M. J., 2016 Comparative idiosyncrasies in life extension by reduced mTOR signalling and its distinctiveness from dietary restriction. Aging Cell 15: 737–743. 10.1111/acel.12489 27139919 PMC4933670

[bib22] Harper J. M. Wilkinson J. E. Miller R. A., 2010 Macrophage migration inhibitory factor-knockout mice are long lived and respond to caloric restriction. FASEB J. 24: 2436–2442. 10.1096/fj.09-152223 20219983 PMC2887269

[bib23] Hayflick L., 2000 The future of ageing. Nature 408: 267–269. 10.1038/35041709 11089985

[bib24] Hinkal G. Parikh N. Donehower L. A., 2009 Timed somatic deletion of p53 in mice reveals age-associated differences in tumor progression. PLoS One 4: e6654 10.1371/journal.pone.0006654 19680549 PMC2721630

[bib25] Hofmann J. W. Zhao X. De Cecco M. Peterson A. L. Pagliaroli L., 2015 Reduced expression of MYC increases longevity and enhances healthspan. Cell 160: 477–488. 10.1016/j.cell.2014.12.016 25619689 PMC4624921

[bib26] Hughes B. G. Hekimi S., 2016 Different mechanisms of longevity in long-lived mouse and Caenorhabditis elegans mutants revealed by statistical analysis of mortality rates. Genetics 204: 905–920. 10.1534/genetics.116.192369 27638422 PMC5105868

[bib27] Jeng Y. M. Cai-Ng S. Li A. Furuta S. Chew H., 2007 Brca1 heterozygous mice have shortened life span and are prone to ovarian tumorigenesis with haploinsufficiency upon ionizing irradiation. Oncogene 26: 6160–6166. 10.1038/sj.onc.1210451 17420720

[bib28] Kang S. Louboutin J. P. Datta P. Landel C. P. Martinez D., 2013 Loss of HtrA2/Omi activity in non-neuronal tissues of adult mice causes premature aging. Cell Death Differ. 20: 259–269. 10.1038/cdd.2012.117 22976834 PMC3554338

[bib29] Kenyon C. J., 2010 The genetics of ageing. Nature 464: 504–512 (erratum: Nature 467: 622). 10.1038/nature08980 20336132

[bib30] Keyes W. M. Wu Y. Vogel H. Guo X. Lowe S. W., 2005 p63 deficiency activates a program of cellular senescence and leads to accelerated aging. Genes Dev. 19: 1986–1999. 10.1101/gad.342305 16107615 PMC1199570

[bib72] Ladiges W. Van Remmen H. Strong R. Ikeno Y. Treuting P., 2009 Lifespan extension in genetically modified mice. Aging Cell 8: 346–352. 19485964 10.1111/j.1474-9726.2009.00491.x

[bib31] Lapointe J. Stepanyan Z. Bigras E. Hekimi S., 2009 Reversal of the mitochondrial phenotype and slow development of oxidative biomarkers of aging in long-lived Mclk1+/− mice. J. Biol. Chem. 284: 20364–20374. 10.1074/jbc.M109.006569 19478076 PMC2740461

[bib32] Laurent G. Solari F. Mateescu B. Karaca M. Castel J., 2008 Oxidative stress contributes to aging by enhancing pancreatic angiogenesis and insulin signaling. Cell Metab. 7: 113–124. 10.1016/j.cmet.2007.12.010 18249171

[bib33] Li H. Vogel H. Holcomb V. B. Gu Y. Hasty P., 2007 Deletion of Ku70, Ku80, or both causes early aging without substantially increased cancer. Mol. Cell. Biol. 27: 8205–8214. 10.1128/MCB.00785-07 17875923 PMC2169178

[bib34] Li Q. Ren J., 2007 Influence of cardiac-specific overexpression of insulin-like growth factor 1 on lifespan and aging-associated changes in cardiac intracellular Ca2+ homeostasis, protein damage and apoptotic protein expression. Aging Cell 6: 799–806. 10.1111/j.1474-9726.2007.00343.x 17973971

[bib35] Liu X. Jiang N. Hughes B. Bigras E. Shoubridge E., 2005 Evolutionary conservation of the clk-1-dependent mechanism of longevity: loss of mclk1 increases cellular fitness and lifespan in mice. Genes Dev. 19: 2424–2434. 10.1101/gad.1352905 16195414 PMC1257397

[bib36] Markovich D. Ku M. C. Muslim D., 2011 Increased lifespan in hyposulfatemic NaS1 null mice. Exp. Gerontol. 46: 833–835. 10.1016/j.exger.2011.05.008 21651971

[bib37] Matheu A. Maraver A. Klatt P. Flores I. Garcia-Cao I., 2007 Delayed ageing through damage protection by the Arf/p53 pathway. Nature 448: 375–379. 10.1038/nature05949 17637672

[bib38] Meira L. B. Calvo J. A. Shah D. Klapacz J. Moroski-Erkul C. A., 2014 Repair of endogenous DNA base lesions modulate lifespan in mice. DNA Repair (Amst.) 21: 78–86. 10.1016/j.dnarep.2014.05.012 24994062 PMC4125484

[bib39] Min J. N. Whaley R. A. Sharpless N. E. Lockyer P. Portbury A. L., 2008 CHIP deficiency decreases longevity, with accelerated aging phenotypes accompanied by altered protein quality control. Mol. Cell. Biol. 28: 4018–4025. 10.1128/MCB.00296-08 18411298 PMC2423116

[bib40] Murga M. Bunting S. Montana M. F. Soria R. Mulero F., 2009 A mouse model of ATR-Seckel shows embryonic replicative stress and accelerated aging. Nat. Genet. 41: 891–898. 10.1038/ng.420 19620979 PMC2902278

[bib41] Muro A. F. Chauhan A. K. Gajovic S. Iaconcig A. Porro F., 2003 Regulated splicing of the fibronectin EDA exon is essential for proper skin wound healing and normal lifespan. J. Cell Biol. 162: 149–160. 10.1083/jcb.200212079 12847088 PMC2172721

[bib42] Ortega-Molina A. Efeyan A. Lopez-Guadamillas E. Munoz-Martin M. Gomez-Lopez G., 2012 Pten positively regulates brown adipose function, energy expenditure, and longevity. Cell Metab. 15: 382–394. 10.1016/j.cmet.2012.02.001 22405073

[bib43] Pletcher S. D., 2002 Mitigating the tithonus error: genetic analysis of mortality phenotypes. Sci. SAGE KE 2002: pe14. 14603004 10.1126/sageke.2002.37.pe14

[bib44] Pletcher S. D. Khazaeli A. A. Curtsinger J. W., 2000 Why do life spans differ? Partitioning mean longevity differences in terms of age-specific mortality parameters. J. Gerontol. A Biol. Sci. Med. Sci. 55: B381–B389. 10.1093/gerona/55.8.B381 10952359

[bib45] Pyo J. O. Yoo S. M. Ahn H. H. Nah J. Hong S. H., 2013 Overexpression of Atg5 in mice activates autophagy and extends lifespan. Nat. Commun. 4: 2300 10.1038/ncomms3300 23939249 PMC3753544

[bib46] Ran Q. Liang H. Ikeno Y. Qi W. Prolla T. A., 2007 Reduction in glutathione peroxidase 4 increases life span through increased sensitivity to apoptosis. J. Gerontol. A Biol. Sci. Med. Sci. 62: 932–942. 10.1093/gerona/62.9.932 17895430

[bib47] Razzaque M. S. Sitara D. Taguchi T. St-Arnaud R. Lanske B., 2006 Premature aging-like phenotype in fibroblast growth factor 23 null mice is a vitamin D-mediated process. FASEB J. 20: 720–722. 10.1096/fj.05-5432fje 16436465 PMC2899884

[bib48] Satoh A. Brace C. S. Rensing N. Cliften P. Wozniak D. F., 2013 Sirt1 extends life span and delays aging in mice through the regulation of Nk2 homeobox 1 in the DMH and LH. Cell Metab. 18: 416–430. 10.1016/j.cmet.2013.07.013 24011076 PMC3794712

[bib49] Schriner S. E. Linford N. J. Martin G. M. Treuting P. Ogburn C. E., 2005 Extension of murine life span by overexpression of catalase targeted to mitochondria. Science 308: 1909–1911. 10.1126/science.1106653 15879174

[bib50] Selman C. Tullet J. M. Wieser D. Irvine E. Lingard S. J., 2009 Ribosomal protein S6 kinase 1 signaling regulates mammalian life span. Science 326: 140–144. 10.1126/science.1177221 19797661 PMC4954603

[bib51] Sibille E. Su J. Leman S. Le Guisquet A. M. Ibarguen-Vargas Y., 2007 Lack of serotonin1B receptor expression leads to age-related motor dysfunction, early onset of brain molecular aging and reduced longevity. Mol. Psychiatry 12: 1042–1056. 10.1038/sj.mp.4001990 17420766 PMC2515886

[bib52] Streeper R. S. Grueter C. A. Salomonis N. Cases S. Levin M. C., 2012 Deficiency of the lipid synthesis enzyme, DGAT1, extends longevity in mice. Aging (Albany N.Y.) 4: 13–27. 10.18632/aging.100424 PMC3292902 22291164

[bib53] Sun L. Q. Lee D. W. Zhang Q. Xiao W. Raabe E. H., 2004 Growth retardation and premature aging phenotypes in mice with disruption of the SNF2-like gene, PASG. Genes Dev. 18: 1035–1046. 10.1101/gad.1176104 15105378 PMC406293

[bib54] Sun L. Y. Spong A. Swindell W. R. Fang Y. Hill C., 2013 Growth hormone-releasing hormone disruption extends lifespan and regulates response to caloric restriction in mice. Elife 2: e01098 10.7554/eLife.01098 24175087 PMC3810783

[bib55] Tacutu R. Craig T. Budovsky A. Wuttke D. Lehmann G., 2013 Human ageing genomic resources: integrated databases and tools for the biology and genetics of ageing. Nucleic Acids Res. 41: D1027–D1033. 10.1093/nar/gks1155 23193293 PMC3531213

[bib56] Taguchi A. Wartschow L. M. White M. F., 2007 Brain IRS2 signaling coordinates life span and nutrient homeostasis. Science 317: 369–372. 10.1126/science.1142179 17641201

[bib57] Tocchetti A. Soppo C. B. Zani F. Bianchi F. Gagliani M. C., 2010 Loss of the actin remodeler Eps8 causes intestinal defects and improved metabolic status in mice. PLoS One 5: e9468 10.1371/journal.pone.0009468 20209148 PMC2830459

[bib58] Tomás-Loba A. Flores I. Fernández-Marcos P. J. Cayuela M. L. Maraver A., 2008 Telomerase reverse transcriptase delays aging in cancer-resistant mice. Cell 135: 609–622. 10.1016/j.cell.2008.09.034 19013273

[bib59] Vakhrusheva O. Smolka C. Gajawada P. Kostin S. Boettger T., 2008 Sirt7 increases stress resistance of cardiomyocytes and prevents apoptosis and inflammatory cardiomyopathy in mice. Circ. Res. 102: 703–710. 10.1161/CIRCRESAHA.107.164558 18239138

[bib60] Vogel H. Lim D. S. Karsenty G. Finegold M. Hasty P., 1999 Deletion of Ku86 causes early onset of senescence in mice. Proc. Natl. Acad. Sci. USA 96: 10770–10775. 10.1073/pnas.96.19.10770 10485901 PMC17958

[bib61] Wang C. H. Chen Y. F. Wu C. Y. Wu P. C. Huang Y. L., 2014 Cisd2 modulates the differentiation and functioning of adipocytes by regulating intracellular Ca2+ homeostasis. Hum. Mol. Genet. 23: 4770–4785. 10.1093/hmg/ddu193 24833725

[bib62] Wang L. Yang L. Debidda M. Witte D. Zheng Y., 2007 Cdc42 GTPase-activating protein deficiency promotes genomic instability and premature aging-like phenotypes. Proc. Natl. Acad. Sci. USA 104: 1248–1253. 10.1073/pnas.0609149104 17227869 PMC1783128

[bib63] Wei K. Clark A. B. Wong E. Kane M. F. Mazur D. J., 2003 Inactivation of Exonuclease 1 in mice results in DNA mismatch repair defects, increased cancer susceptibility, and male and female sterility. Genes Dev. 17: 603–614. 10.1101/gad.1060603 12629043 PMC196005

[bib64] Wu C. Y. Chen Y. F. Wang C. H. Kao C. H. Zhuang H. W., 2012 A persistent level of Cisd2 extends healthy lifespan and delays aging in mice. Hum. Mol. Genet. 21: 3956–3968. 10.1093/hmg/dds210 22661501

[bib65] Wu J. J. Liu J. Chen E. B. Wang J. J. Cao L., 2013 Increased mammalian lifespan and a segmental and tissue-specific slowing of aging after genetic reduction of mTOR expression. Cell Rep. 4: 913–920. 10.1016/j.celrep.2013.07.030 23994476 PMC3784301

[bib66] Yan L. Vatner D. E. O’Connor J. P. Ivessa A. Ge H., 2007 Type 5 adenylyl cyclase disruption increases longevity and protects against stress. Cell 130: 247–258. 10.1016/j.cell.2007.05.038 17662940

[bib67] Yen K. Steinsaltz D. Mobbs C. V., 2008 Validated analysis of mortality rates demonstrates distinct genetic mechanisms that influence lifespan. Exp. Gerontol. 43: 1044–1051. 10.1016/j.exger.2008.09.006 18832022

[bib68] Zhang G. Li J. Purkayastha S. Tang Y. Zhang H., 2013 Hypothalamic programming of systemic ageing involving IKK-beta, NF-kappaB and GnRH. Nature 497: 211–216. 10.1038/nature12143 23636330 PMC3756938

[bib69] Zhang Y. Padalecki S. S. Chaudhuri A. R. De Waal E. Goins B. A., 2007 Caspase-2 deficiency enhances aging-related traits in mice. Mech. Ageing Dev. 128: 213–221. 10.1016/j.mad.2006.11.030 17188333 PMC1828128

[bib70] Zhang Y. Xie Y. Berglund E. D. Coate K. C. He T. T., 2012 The starvation hormone, fibroblast growth factor-21, extends lifespan in mice. Elife 1: e00065 10.7554/eLife.00065 23066506 PMC3466591

[bib71] Zheng S. Clabough E. B. Sarkar S. Futter M. Rubinsztein D. C., 2010 Deletion of the huntingtin polyglutamine stretch enhances neuronal autophagy and longevity in mice. PLoS Genet. 6: e1000838 10.1371/journal.pgen.1000838 20140187 PMC2816686

